# *De novo* assembly, characterization and annotation for the transcriptome of *Sarcocheilichthys sinensis*

**DOI:** 10.1371/journal.pone.0171966

**Published:** 2017-02-14

**Authors:** Chuankun Zhu, Zhengjun Pan, Hui Wang, Guoliang Chang, Nan Wu, Huaiyu Ding

**Affiliations:** 1 Jiangsu Engineering Laboratory for Breeding of Special Aquatic Organisms, Huaiyin Normal University, Huai’an, China; 2 Jiangsu Collaborative Innovation Center of Regional Modern Agriculture & Environmental Protection, Huaiyin Normal University, Huai’an, China; Kumamoto University, JAPAN

## Abstract

The Chinese lake gudgeon *Sarcocheilichthys sinensis* is a small cyprinid fish with great aquaculture potential both for its edible and ornamental values. Nevertheless, available genomic and transcriptomic information for this fish is extremely deficient. In this study, a normalized cDNA library was constructed using 13 mixed tissues of an adult male *S*. *sinensis*, and was sequenced by the Illumina HiSeq2500 platform. *De novo* assembly was performed using 38,911,511 obtained clean reads, and a total of 147,282 unigenes with an average length of 900 bp were finally achieved. 96.2% of these unigenes were annotated in 9 public databases, and 16 segments of growth-related genes were identified for future studies. In addition, 28,493 unigenes were assigned to 61 subcategories of Gene Ontology (GO), and 10,483 unigenes were assigned to 25 categories of Cluster of Orthologous Group (COG). Moreover, 14,943 unigenes were classified into 225 pathways of the Kyoto Encyclopedia of Genes and Genomes (KEGG) database. A total of 30,666 microsatellites were detected from 17,627 unigenes with an average distribution density of 1:2405 bp. This transcriptome data set will be valuable for researches on discovery, expression and evolution on genes of interest. Meanwhile, the identified microsatellites would be useful tools for genetic and genomic studies in *S*. *sinensis*.

## Introduction

With the development of high-throughput sequencing technologies, genetic and genomic information is much easier to obtain for non-model organisms than ever before. Transcriptome analysis is an efficient method for genome survey, massive functional gene identification and molecular marker isolation [1]. Compared to genomic sequences, transcriptomic sequences are all coding DNA and have a much higher rate of functional information; therefore, they are more helpful in revealing molecular mechanisms of functional genes [2,3]. RNA sequencing (RNA-Seq), such as Illumina, Roche 454 and Solexa, is a powerful high-throughput sequencing approach, and has been applied to acquire massive of transcriptomic information in aquatic animals. Some of these studies were on characterization and annotation of transcriptomes [4–7], and others focused on varies fields including development of simple sequence repeat markers (SSRs) and single nucleotide polymorphism markers (SNPs) [8–10], isolation of immune-related genes [11–13], evolution analyses [14,15], adverse environment tolerance [16,17], sex determination [18,19], and so on.

Cyprinidae is believed to be the largest fish family with the greatest species richness in East Asia, and nearly 1/3 cyprinids are naturally distributed in China [20]. The Chinese lake gudgeon *Sarcocheilichthys sinensis* (Bleeker, 1871) is a small benthic freshwater fish belonging to the subfamily Gobioninae of Cyprinidae, and it widely distributes in rivers and lakes of flat areas in China [21]. Since *S*. *sinensis* has delicious taste and rich nutrition, it is preferred by many Chinese consumers; moreover, because of its colorful appearance, it is also popular among ornamental fish lovers recently [22]. Although aquaculture of this fish has been implemented in some areas of China, the relatively limited production could not satisfy the increasing market requirements. Therefore, persistent capture pressures were borne on wild populations resulting in the sharp decline of its natural resources in recent years [22,23].

Given the current situation of *S*. *sinensis*, studies on this fish are increasing presently. However, most of these studies focused on the reproduction and aquaculture of this fish [22–24]; researches on germplasm estimation, population diversity and structure, genetic conservation and protection were seldom. The most probable reason may be the lack of its available genetic and genomic information. Although the complete mitochondrial genome has been reported [25–27], available sequences in *S*. *sinensis* were still limited compared to other Gobioninae fish species, such as *Gobiocypris rarus* [28], *Hemibarbus maculates* [29], *Coreius heterodon* [30], etc.

In this study, the Illumina platform was employed to sequence a cDNA library constructed using 13 mixed tissues to obtain the transcriptome information for *S*. *sinensis*. The information obtained in this study would provide valuable resources for further studies on functional gene analyses, population structure estimation, germplasm conservation, genomic evolution, genetic linkage map construction, quantitative trait loci (QTL) identification and marker-assisted selection (MAS) breeding in *S*. *sinensis*.

## Materials and methods

### Ethics statement

Usage of *Sarcocheilichthys sinensis* was permitted by Managing Committee of Huai’an Fisheries Technical Guidance Station. All the experimental animal programs applied in this study were approved by the Huaiyin Normal University’s Animal Care and Use Committee (HNUACUC), and followed the experimental basic principles. Experimental fish was split after being anaesthetized by MS222 to sample different tissues. All efforts were made to minimize suffering of the experimental fish.

### Sample preparation and RNA extraction

A male *S*. *sinensis* which was caught from the Hongze Lake by members of Huai’an Fisheries Technical Guidance Station was used in this study. After anaesthesia, thirteen tissues including skin, muscle, eye, brain, hypothalamus, pituitary, heart, liver, spleen, kidney, intestines, gill and testicle were sampled and immediately placed in liquid nitrogen to freeze and then stored at -80°C until use.

For RNA extraction, approximately 10 mg of each tissue was put in a 2 mL tube, then 500 μL of liquid nitrogen was put into the tube and tissues were shattered and mixed using a Pro200 tissue homogenizer (Pro, USA). After volatilization of liquid nitrogen, RNA was extracted following the manufacturers’ instructions of TRIzol Reagent (Invitrogen, USA). Extracted RNA was treated by DNase I (Takara, Japan) at 37°C for 45 min to remove residual DNA. Quality of RNA was verified through Nanodrop 2000, Aglient 2100 and Qubit 2.0 Bioanalyzers.

### Library construction and Illumina sequencing

The Magnetic Oligo (dT) Beads (Invitrogen, USA) was applied to isolate poly (A) mRNA from total RNA. And the mRNA was randomly fragmented by the fragmentation buffer. Using these fragments as templates, cDNA was synthesized and then purified using AMPure XP beads (Beckman, USA). After end reparation and single nucleotide A (adenine) addition for the purified cDNA, adapters were connected. Suitable fragments were selected by AMPure XP beads as templates for PCR amplification and the library was then obtained. After that Agilent 2100 Bioanaylzer and ABI StepOnePlus Real-Time PCR System were applied for quantification and qualification of the library, respectively. Finally, high-throughput sequencing was conducted through the Illumina HiSeq 2500 platform at Biomarker Technologies Co., Ltd., Beijing, China according to the manufacturer’s instructions (Illumina, San Diego, CA, USA) to generate 125-bp paired-end reads.

### Data processing and *de novo* assembly

Raw reads were trimmed by SeqPrep (https://github.com/jstjohn/SeqPrep) and Condetri_v2.0.pl (http://code.google.com/p/condetri/downloads/detail?name=condetri_v2.0.pl) softwares to discard dirty reads including adaptor sequences, highly redundant sequences, reads containing more than 10% ambiguous bases, and low quality reads with abase quality of less than 20 (Q-value <20). After this, high-quality clean reads were obtained and *de novo* assembly was implemented using the Trinity software [31] with default settings. Briefly, reads of a certain length of overlap were combined to form contigs, and then reads were mapped back to contigs. After that contigs were clustered into transcripts, and redundancies in these transcripts were then removed to obtain unigenes.

### Annotation of unigenes

Assembled unigenes were aligned against the NCBI non-redundant nucleotide sequence database (Nt) by BLASTn with an E-value cut off of 10^−5^. Then they were searched in other public databases including Nr (non-redundant protein database, NCBI), GO (Gene Ontology, http://www.geneontology.org/), Pfam (Protein family, http://pfam.xfam.org/), Swiss-Prot (http://www.uniprot.org/), TrEMBL (Translations of The European Molecular Biology Laboratory nucleotide sequence entries, http://www.bioinfo.pte.hu/more/TrEMBL.htm), COG (Clusters of Orthologous Groups, http://www.ncbi.nlm.nih.gov/COG/), KOG (Eukaryotic Ortholog Groups, http://www.ncbi.nlm.nih.gov/KOG/) and KEGG (Kyoto Encyclopedia of Genes and Genomes, http://www.genome.jp/kegg/) through BLASTx under the same criterion as BLASTn. For Nr annotation, the program Blast2GO [32] was applied to predict GO terms that unigenes related to, then the WEGO software [33] was employed to classify GO functions for all unigenes and analyze distribution of gene functions in *S*. *sinensis* at the macro level. In addition, CDS (Coding sequences) for unigenes were predicted using the software Getorf (http://emboss.sourceforge.net/apps/cvs/emboss/apps/getorf.html).

In order to test the reliability of this transcriptome for deriving interested genes, 10 mRNA sequences of growth-related genes in zebrafish were applied, which were growth hormone (*gh*) (NM_001020492), growth hormone receptor (*ghr*) (NM_001083578 and NM_001111081), somatostatin (*ss*) (NM_183070 and NM_001045431), insulin-like growth factor (*igf*) (NM_131825, NM_131433 and NM_001001815) and myostatin (*mstn*) (NM_001004122 and NM_131019). All of these sequences were used to search homologous segments in the transcriptome of *S*. *sinensis* using local BLAST with the E-value criterion of 10^−5^.

Comparisons between *S*. *sinensis* and zebrafish on these 10 genes were done using translated amino acid sequences of them, which were deduced by applying the online software Sequence Manipulation Suite (http://www.bio-soft.net/sms/). Furthermore, coding mRNA sequences for 8 of these 10 genes from different subfamilies (mainly Cyprininae, Hypophthalmichthyinae, Schizothoracinae, Leuciscinae, Cultrinae, Gobioninae and Danioninae) in Cyprinidae were used to perform phylogenetic analysis with homologous genes of *S*. *sinensis*, and gene sequences of Japanese flounder (*Paralichthys olivaceus*) were applied as outgroups. Sequence alignments were performed by ClustalX 2.0 (http://www.clustal.org/clustal2/), and phylogenetic trees were constructed using MEGA 5.0 (http://www.megasoftware.net/) via the Neighbor-Joining (N-J) method based on the Poisson-corrected distances with 10,000 bootstraps.

### Microsatellite detection

In order to understand distributions of microsatellites (also known as SSRs) and to develop new markers in the transcriptome of *S*. *sinensis*, the program MISA (http://pgrc.ipk-sgatersleben.de/misa/) was used to detect microsatellite repeat motifs for each unigene with a length of more than 1,000 bp. The minimum repeat time for core repeat motifs was set to ten for mono-nucleotide, six for di- nucleotides, and five for tri-, tetra-, penta- and hexa- nucleotides. Primers for these microsatellites were designed by Primer 3 [34], and parameters were set as follows: lengths of primers were 20–25 bases with an optimum of 22 bases; PCR product sizes ranged from 100 to 250 bp; and optimum annealing temperature varied from 50°C to 60°C; other parameters were set as default values.

## Results

### Illumina sequencing and *de novo* assembly

After trimming and quality filtration of raw data, a total of 38,911,511 clean reads containing 9,727,877,750 clean nucleotides were generated with an average length of 250 bp. All of these clean reads have been submitted to the Sequence Read Archive database at NCBI (accession no: SRR4242076). The average GC content of the clean reads was 46.43%, and the proportion of nucleotides with quality value larger than 30 in reads (Q30) was 87.42%.

**Fig 1:**
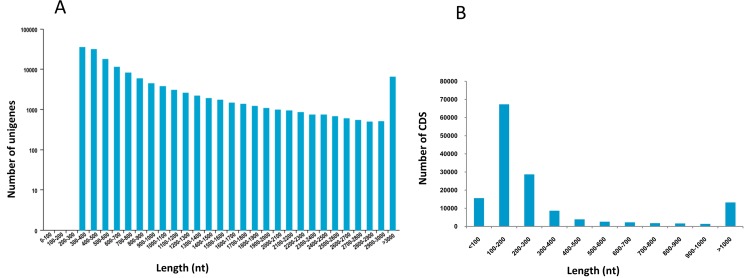
Overview of the transcriptome assembly for *Sarcocheilichthys sinensis*. (A) Size distribution of unigenes; (B) Size distribution of coding sequences (CDS).

A total of 194,930 transcripts (224,384,594 nucleotides in total) were obtained by assembling clean reads using the Trinity program, with an average length of 1,151 bp and an N50 length of 1,924 bp (Table 1). All transcripts were more than 300 bp in length with 61.7% of which being longer than 500 bp. The transcripts were further clustered and assembled into 147,282 unigenes. And the unigenes were all longer than 300 bp with the average and N50 lengths of 900 bp and 1,204 bp, respectively (Table 1). Of the 147,282 unigenes, 55.1% (81,170) were longer than 500 bp, and 22.8% (33,496) were longer than 1 kb (Table 1 and Fig 1A). In addition, CDS ranging from 30 to 26,388 bp with an average length of 387 bp were predicted in 147,188 (99.9%) of these unigenes (S1 Table). Most CDS have a length ranged from 100 to 200 bp (67,296, 45.7%), and 43.7% (64,301) were longer than 200 bp including 13,208 (9.0%) ones longer than 1 kb (Fig 1B).

**Table 1:** Statistical summary of the *de novo* transcriptome assembly for *Sarcocheilichthys sinensis*.

Length range (bp)	Transcript	Unigene
300–500	74,663	66,112
500–1000	57,329	47,674
1000–2000	32,546	20,098
2000+	30,392	13,398
Total number	194,930	147,282
Total length	224,384,594	132,493,261
N50 length	1,924	1,204
Mean length	1,151	900

### Functional annotation of unigenes

**Fig 2:**
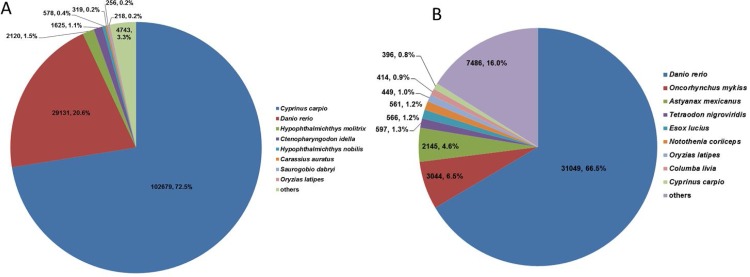
Species distribution of homologies for *Sarcocheilichthys sinensis*. (A) Overall species distribution of the top BLAST hits against available public databases; (B) Species distribution of homologies against the Nr database.

Results of functional annotation showed that 141,669 (96.2%) of the 147,282 unigenes were annotated against databases of Nt, Nr, COG, GO, KEGG, KOG, Pfam, Swissprot and TrEMBL, among which Nt contained the most homologies (Table 2, S2 Table). These homologies distributed in genomes of many fish species, out of which common carp (*Cyprinus carpio*) had the most homologies constituting 72.5% (102,679) of the annotated unigenes, followed by zebrafish of 20.6% (29,131) (Fig 2A). While in the database Nr, annotation rates for unigenes were the most in zebrafish (*Danio rerio*) of 66.5% (Fig 2B). Annotated sequences for unigenes were all longer than 300 bp with 33,462 (23.6%) of which being longer than 1 kb (Table 2). Additionally, the rest 5,613 (3.8%) unigenes had no BLAST hits in these databases, indicating that they might contain novel genes with unknown functions.

**Table 2:** Summary of functional annotations for unigenes of *Sarcocheilichthys sinensis*.

Annotated Database	Annotated Number	300< = length<1000 (bp)	length> = 1000 (bp)
COG Annotation	10,483	3,142	7,341
GO Annotation	28,493	11,281	17,212
KEGG Annotation	14,943	5,405	9,538
KOG Annotation	26,326	9,727	16,599
Pfam Annotation	28,205	8,579	19,626
Swissprot Annotation	27,272	9,689	17,583
TrEMBL Annotation	47,576	23,533	24,043
Nr Annotation	47,248	23,222	24,026
Nt Annotation	140,308	106,904	33,404
All Annotated	141,669	108,207	33,462

**Fig 3:**
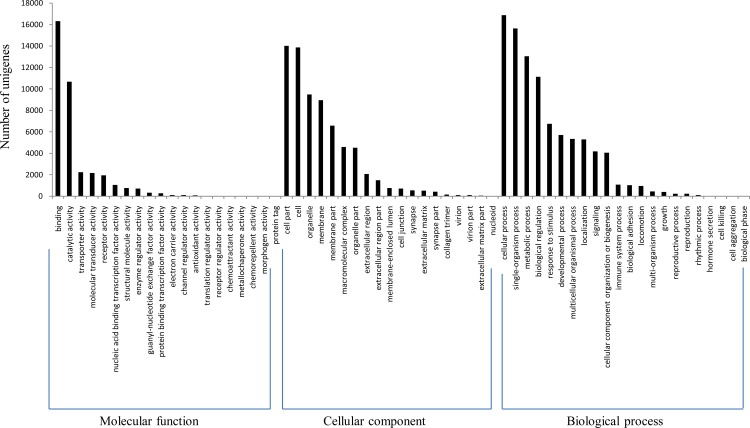
Gene Ontology (GO) classification of assembled unigenes.

The program Blast2GO was utilized for classification of the predicted functions of unigenes, which were classified into three categories: cellular component, molecular function and biological process. The category “biological process” consisting of 22 functional groups showed the highest number of annotations with cellular process being the dominant group (18.3%), followed by single-organism process (16.9%) (Fig 3, S3 Table). The “cellular component” category contained 19 functional groups with most unigenes being related to terms of cell part (20.4%) and cell (20.2%) (Fig 3, S3 Table). For the category of “molecular function”, 20 functional groups were predicted with binding (44.5%) and catalytic activity (29.1%) being dominant terms (Fig 3, S3 Table).

**Fig 4:**
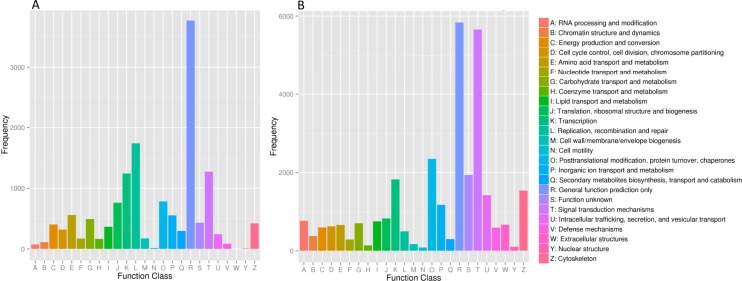
Functional classification of unigenes. (A) COG (Clusters of Orthologous Groups) functional classification of unigenes; (B) KOG (Eukaryotic Ortholog Groups) functional classification of unigenes.

A total of 10,483 unigenes were annotated in the COG database and classified into 25 COG classifications with term abbreviation ranged from A to Z. Among these terms the term R (general function prediction only) gathered the most number of unigenes, followed by L (Replication, recombination and repair) (Fig 4A). Furthermore, 26,326 unigenes were annotated in the KOG database and clustered into 25 KOG categories with “general function prediction only” (abbreviated as R) containing the greatest number of unigenes, followed by “signal transduction mechanism” (abbreviated as T) (Fig 4B). Additionally, 14,943 unigenes were annotated in the KEGG database and assigned to 225 KEGG pathways with “MAPK signaling pathway” owning the most annotated unigenes (S4 Table).

Through local BLAST search in the *S*. *sinensis* transcriptome, a total of 16 sequences that were homologous with 10 growth-related zebrafish genes were isolated (S5 Table). The results showed that four zebrafish genes owned more than one homologies (S5 Table), which may be caused by two reasons according to our analysis. One reason was that the homology of a given zebrafish gene was divided into different fragments by missing nucleotides, such as *ghra*; and the other reason was the existence of polymorphic sites among homologies of one gene, such as *igf2b* (S1 File). Amino acid sequence comparison between *S*. *sinensis* and zebrafish showed that *ss3* was the most conserved gene with a variation rate of 1.8%, while *ghra* was the least conserved one with a variation rate of 30.1% (S1 Fig). In addition, variation rates were also different between subtypes of genes, for instance, the rate in *igf2a* (16.5%) was much higher than that in *igf1* (5.0%) and *igf2b* (2.0%) (S1 Fig). N-J trees showed that *S*. *sinensis* genes of *igf*, *mstn*, *gh* and *ghr* were all clustered with those of other cyprinids, and subtypes of *igf*, *mstn* and *ghr* were all clustered with corresponding sequences from other cyprinid species (S2 Fig). Moreover, *igf* and *mstn* of the outgroup species *Paralichthys olivaceus* were all gathered with their homologous genes in cyprinids instead of forming an outgroup branch, however, *ghr* of *P*. *olivaceus* separated clearly with those of cypinid species (S2 Fig).

### Microsatellite identification

In order to obtain microsatellites containing flanking sequences with enough lengths for primer design, only those unigenes with a length of more than 1 kb were used for SSR detection. Using the software MISA, 33,496 unigenes (total length of 73,738,473 bp) were screened out for microsatellite identification, and SSRs were finally detected in 17,627 of them. A total of 30,666 SSRs were detected (S6 Table), and according to the total length of the 33,496 unigenes, the average distribution density of SSRs was calculated to be 1:2,405 bp with an average SSR frequency of 0.21 (30,666/147,282) throughout the transcriptome of *S*. *sinensis*. Of the17,627 unigenes containing SSRs, 7,794 owned more than one SSR with a percentage of 44.2%. Excluding those containing mononucleotide SSRs, Nt-annotation for 12,410 SSR-containing unigenes were conducted with 5,726 of which being successfully annotated (S6 Table).

Among the identified 30,666 SSRs, repeats with mononucleotide motifs were the most abundant (18007, 58.7%), followed by di-nucleotide (9295, 30.3%) (Table 3). A total of 72 types of repeat motifs were found in the *S*. *sinensis* transcriptome. The most abundant motif was A/T (17,565, 57.3%), followed by AC/GT (5,591, 18.2%) and AG/CT (2,292, 7.5%) (Table 3, S7 Table). For SSRs with tri-, tetra-, penta- and hexa-nucleotide motifs, the most abundant types were AAT/ATT (646, 2.1%), AGAT/ATCT (98, 0.3%), AAGAG/CTCTT (8, 0.03%) and ACACTC/AGTGTG (6, 0.02%), respectively (Table 3, S7 Table).

**Table 3:** Summary of SSRs identified from the transcriptome of *Sarcocheilichthys sinensis*.

SSR Type	Number	Percentage	Dominant motif	Number of Dominant motif	Percentage of dominant motif
Mononucleotide	18,007	58.72%	A/T	17,565	57.30%
Dinucleotide	9,295	30.31%	AC/GT	5,591	18.20%
Trinucleotide	2,633	8.59%	AAT/ATT	646	2.10%
Tetranucleotide	656	2.14%	AGAT/ATCT	98	0.30%
Pentanucleotide	55	0.18%	AAGAG/CTCTT	8	0.03%
Hexanucleotide	20	0.07%	ACACTC/AGTGTG	6	0.02%
Total	30,666	100%	-	23,914	77.95%

Repeat times of these SSR motifs ranged from 5 to 97. Most SSR motifs repeated more than 15 times with a percentage of 21.5% (6,592), and repeat times of 10 (5,343, 17.4%) and 11 (3,723, 12.1%) were also common (Table 4). Excluding mononucleotide types, copy numbers for most SSRs were from 5 to 10 (9,612, 75.9%), only a small percent were more than 15 repeat times (1,177, 9.3%) (Table 4). Finally, 54,321 primer pairs (3 pairs for each SSR) were designed for 18,117 SSRs which have enough flanking sequence lengths (S8 Table).

**Table 4:** Summary of different repeat times for SSRs isolated from the transcriptome of *Sarcocheilichthys sinensis*.

	5	6	7	8	9	10	11	12	13	14	15	>15	Total
Mononucleotide	0	0	0	0	0	4,666	3,003	1,905	1,333	944	741	5,415	18,007
Dinucleotide	0	2,569	1,554	960	756	618	669	542	166	206	143	1,112	9,295
Trinucleotide	1,286	592	330	263	27	44	37	16	14	4	4	16	2,633
Tetranucleotide	271	197	33	23	19	11	11	13	13	6	12	47	656
Pentanucleotide	22	4	6	3	5	2	1	1	6	3	0	2	55
Hexanucleotide	5	2	6	1	1	2	2	1	0	0	0	0	20
Total	1,584	3,364	1,929	1,250	808	5,343	3,723	2,478	1,532	1,163	900	6,592	30,666
Percentage	5.17%	10.97%	6.29%	4.08%	2.63%	17.42%	12.14%	8.08%	5.00%	3.79%	2.93%	21.50%	100%

## Discussion

### Transcriptome sequencing and *de novo* assembly

In order to obtain sequences of expressed genes in adult male *S*. *sinensis* as many as possible, 13 mixed tissues were used for library construction and sequencing. The data size (9.73 Gb) obtained in this study was larger than that of most reported cyprinid fishes including common carp (7.39 Gb) [35], grass carp (5.38 Gb) [36], blunt snout bream *Megalobrama amblycephala* (4.62 Gb) [37], yellow-cheek carp *Elopichthys bambusa* (2.67 Gb) [15], etc. The relatively larger data size in this study may because that this library was constructed using 13 mixed tissues, while in other fishes, single or a few mixed tissues with relatively fewer expressed genes were used. As larger data size could increase the coverage depth of the actual transcriptome, therefore, these data could facilitate the accuracy and reliability of *de novo* assembly in *S*. *sinensis*.

Through application of the software Trinity, 94,930 transcripts were assembled out with an average length of 1,151 bp, which was longer than that of many cyprinids such as topmouth culter *Erythroculter ilishaeformis* (593 bp) [38], naked carp *Gymnocypris przewalskii* (952 bp) [13] and blunt snout bream (998 bp) [37], while was shorter when compared to common carp (1400.57 bp) [35] and grass carp (1,470 bp) [36]. Moreover, it seemed that the average length of transcripts in this study was longer than that obtained by 454 RNA-Seq platform in most fishes, such as crucian carp *Carassius auratus* (492.6 bp) [4], blunt snout bream (730 bp) [10] and common carp (888 bp) [39].

Similar to previous transcriptome studies using Illumina sequencing in other fishes [13,35,37], 147,282 unigenes were obtained through clustering and assembly of transcripts in *S*. *sinensis*. 22.8% of these unigenes were longer than 1 kb, which was similar to those of blunt snout bream (17.8%) [37] and naked carp (28.35%) [13], and higher than those of Japanese flounder *Paralichthys olivaceus* (14.01%) [12] and Chinese sturgeon *Acipenser sinensis* (15.9%) [40], but was lower than those of common carp (50.04%) [35] and large-scale loach *Paramisgurnus dabryanus* (48.1%) [9].

In this study, CDS were predicted in 99.9% of the assembled unigenes, indicating that assembly of *S*. *sinensis* unigenes was reliable and majority of unigenes were derived from intact protein-coding transcripts. This ratio was much higher than that of 64.9% in common carp [35] and 53.6% in grass carp [36]. Among these CDS, 43.7% (64,301) were longer than 200 bp, which was lower than that of 57.39% in large-scale loach [9], but much higher than that of blunt snout bream (21%) in which transcriptome was sequenced using the Roche 454 platform [10]. In spite of this, these predicted CDS would provide useful information for analyses on genes of interest in future studies of *S*. *sinensis*.

### Function annotation of unigenes

Through BLAST search against 9 databases, 96.2% of *S*. *sinensis* unigenes were successfully annotated, which was much higher than that of many reported fishes including common carp (81.1%) [35], naked carp (73.3%) [13], silver carp (63.2%) [5], grass carp (62.6%) [36] and so on. The reason for the relatively higher annotation ratio of unigenes in *S*. *sinensis* maybe more databases being used for detection of homologies in this study. Of these annotated unigenes, genome of common carp have more homologies than that of zebrafish, which was coincide with the evolutionary relationships among the three fishes that *S*. *sinensis* (Gobioninae) was closer with common carp (Cyprininae) than zebrafish (Danioninae) [41,42].

A total of 28,493 unigenes (19.3%) were assigned into 61 GO subcategories, which was similar to that of many previously reported fishes, including common carp (60 subcategories) [35], grass carp (60 subcategories) [36] and naked carp (62 subcategories) [13]. And the result of COG classification also coincided with that of other fish species [13,35–37]. In addition, the number of KEGG pathways in this study (225) was similar to those in naked carp (252) and silver carp (218), but lower than those in common carp (335), blunt snout bream (315) and grass carp (317). As the later three carps had been treated with pathogens, many immune-related pathways may be stimulated; while *S*. *sinensis*, naked carp and silver carp were not treated, so annotated pathways in these fishes were relatively fewer.

Similarity analyses in the database Nr showed that unigenes of *S*. *sinensis* had the most homologies in cDNA sequences of zebrafish (66.5%) than any other fishes. Compared to fishes that had the highest unigene BLAST hits in zebrafish, this homologous rate was similar to that of large-scale loach (60.9%) [9] and common carp (59.8%) [35], higher than that in silver carp (52.5%) [5], but lower than those in blunt snout bream (86%) [37], naked carp (77.17%) [13] and plateau fish *Triplophysa dalaica* (78.2%) [17]. Many factors including percentages of long unigenes, number of tissues applied for sequencing, evolutionary relationships, etc. could cause these differences of homologous rates. Although 13 tissues were applied in this study, the whole *S*. *sinensis* transcriptome was still not completely covered as some rare transcripts which were not expressed in these tissues may be missed.

Since growth is an important trait for fish aquaculture and breeding, we applied 10 growth-related genes to test the reliability of *S*. *sinensis* transcriptome for deriving interested genes. Fortunately, fragments of the 10 genes were all detected in *S*. *sinensis*, although some of them were not complete cDNA sequences. Comparative analyses of the 10 genes indicated their different conservation levels, which maybe a reflection of diverse natural selection pressures on these genes during evolution [43]. Through the N-J trees of *igf*, *mstn*, *gh* and *ghr*, it is easy to find that *S*. *sinensis* (Gobioninae) is close-related to fishes from Leuciscinae, Hypophthalmichthyinae and Cultrinae than other subfamilies, which was coincided with cyprinid phylogeny reported previously [41,42]. And trees of *igf*, *mstn* and *ghr* indicated that subtypes of genes emerged earlier than divergency of Cyprinidae subfamilies, furthermore, subtypes of *igf* and *mstn* even separated before formation of Cypriniformes and Perciformes, while *ghra* and *ghrb* divided after the separation of the two orders. These results could not only provide useful genes for future studies on growth but also confirm the reliability and accuracy of this transcriptome for discovery of novel genes, mining of genetic markers and further genetic analysis in *S*. *sinensis*.

### Distribution of SSRs in *S*. *sinensis* transcriptome

In total, 30,666 SSRs with different repeat motifs were detected from 147,282 unigenes indicating that each unigene contains 0.21 SSR on average. This SSR frequency was similar to those in large-scale loach (0.21) [9], naked carp (0.15) [13] and silver carp (0.16) [5], higher than those in blunt snout bream (0.07) [37], grass carp (0.05) [36] and crucian carp (0.09) [4], while lower than 0.36 in common carp [35] and 0.42 in Japanese Flounder [12]. The SSR distribution density for *S*. *sinensis* was 1:2.41 kb, which was higher than 1:3.9 kb in common carp [35] and 1:6.99 kb in large-scale loach [9], but lower than 1:1.04 kb in blunt snout bream [37]. Several factors may contribute to the variety of SSR frequencies and distribution densities in different organisms, such as diversity of genome structures or compositions [44], varied dataset sizes, different criteria and parameters for SSR detection [45].

As reported in common carp [35], mononucleotide repeat was also the most abundant SSR type in *S*. *sinensis*. However, as application values of mononucleotide SSRs were relatively lower owing to potential inaccurate sequence information caused by sequencing errors and assembly mistakes [35], this SSR type was usually excluded for characterization and even not considered during SSR detection [5,13,35–37]. Excluding mononucleotide repeats, the most common SSR motif was dinucleotide repeats (73.4%) in transcriptome of *S*. *sinensis*, which was similar to most reported fishes including common carp (57.6%) [35], blunt snout bream (70.9%) [37], naked carp (64.4%) [13] and so on. However, in some organisms such as large-scale loach, trinucleotide was the richest SSR loci repeats [9], indicating that genome composition of this fish may be quite different with *S*. *sinensis*. In vertebrate the most abundant SSR repeat motif is believed to be AC/GT [46],which has already been affirmed in many fish species [4,13,35–37]. Expectedly, in transcriptome of *S*. *sinensis*, the motif AC/GT was also proved to be the most common type. Excluding mono-nucleotide repeats, SSRs with six tandem repeats had the highest frequency of 26.6%, and this rate was similar to that of 29.74% in naked carp [13]. However, in common carp [35] and large-scale loach [9], SSRs with four tandem repeats were the most abundant, which may be a reflection of genetic differences among fish species.

SSRs isolated from transcriptomes are also known as EST-SSRs (EST, expressed sequence tags). As they are closely related to expressed functional genes, these markers are more valuable than genomic ones and have been widely used in genetic and genomic analyses of fishes. Herein, 5,726 SSRs developed in transcriptome of *S*. *sinensis* were annotated with known genes sequences. These EST-SSRs would be useful for future studies on genetic linkage mapping, comparative mapping, genomic evolution, QTL identification and MAS breeding in this fish.

## Conclusions

In summary, using the high-throughput Illumina HiSeq 2500 platform, the transcriptome of 13 mixed tissues was sequenced in *S*. *sinensis*. The data obtained in this study could help us to understand the transcriptome of adult *S*. *sinensis* on overall, and information of unigenes will be valuable for further researches on genes of interest, such as those related to growth, immunity and physiological adaptation. Meanwhile, a large number of EST-SSRs were developed, which would be useful for studies on genetic and genomic studies in *S*. *sinensis* and other fishes that closely related to it.

## Supporting information

S1 Fig Results of homologous analyses between *Sarcocheilichthys sinensis* and zebrafish based on amino acid sequences of 10 growth-related genes. (PPTX)

S2 Fig Neighbor-Joining trees between *Sarcocheilichthys sinensis* and other cyprinid fishes based on coding mRNA sequences of *gh*, *ghr*, *igf* and *mstn* using *Paralichthys olivaceusas* an outgroup. GenBank accession numbers for applied sequences were: JN711124.1, NM_001020492.2, AY707317.1, AY170124.1, M27000.1, JF340470.1, M23439.1, JN711123.1 for *gh*; NM_001083578.1, NM_001111081.1, AY283778.2, GU300104.1, GU300105.1, GU300107.1, GU300108.1, JN896373.1, JN896374.1, XM_016277637.1, AF293417.1, KX082700.1, AB110985.1, AY691177.1 for *ghr*; NM_131825.2, NM_131433.1, NM_001001815.1, AF332865.1, EU051323.1, JQ398497.1, EF062860.1, HM641129.1, D83272.1, HM755899.1, KC470046.1, AY919608.1, AY919609.1, AJ010602.1, AJ010603.1, AF091454.1 for *igf*; NM_001004122.2, NM_131019.5, KM874826.1, KM874827.1, JQ065336.1, JQ065337.1, HQ634244.2, KP277104.1, KP277103.1, FJ482232.1, GU014395.1, GU014396.1, GU014397.1, GU014398.1, DQ412048.1 for *mstn*.
(PPTX)

S1 File Unigene sequences in transcriptome of *Sarcocheilichthys sinensis* that homologous with 10 zebrafish growth-related genes. (PDF)

S1 Table Information of predicted coding sequences (CDS) for unigenes of *Sarcocheilichthys sinensis*. (XLSX)

S2 Table Information of integrated function annotation of unigenes in *Sarcocheilichthys sinensis*. (XLSX)

S3 Table GO classification of *Sarcocheilichthys sinensis* unigenes. (XLSX)

S4 Table KEGG classification for unigenes of *Sarcocheilichthys sinensis*. (XLSX)

S5 Table Homologous detection of 10 zebrafish growth-related genes in *S.sinensis* transcriptome. *: Poly A was excluded.
(XLSX)

S6 Table Basic information of SSRs and annotation of SSR-containing sequences in transcriptome of *S. sinensis*. a: Mononucleotide repeat SSRs were excluded; p2-p6 stand for di- to hexa-nucleotide motifs respectively; c stand for compound types. *: Indicating SSR sequences that Primers couldn't be designed.
(XLSX)

S7 Table Frequency of different SSR types in transcriptome of *Sarcocheilichthys sinensis*. (XLSX)

S8 Table Information of primers designed for SSRs developed from the transcriptome of *Sarcocheilichthys sinensis*. (XLSX)
