# Supplementary material for: De novo assembly, characterization and annotation for the transcriptome of Sarcocheilichthys sinensis
Source: PLoS One. 2017 Feb 14;12(2):e0171966. doi: 10.1371/journal.pone.0171966 (PMC5308828; doi:10.1371/journal.pone.0171966)
Supplement: S1 Fig — (PPTX) [file pone.0171966.s001.pptx]

## Slide 1
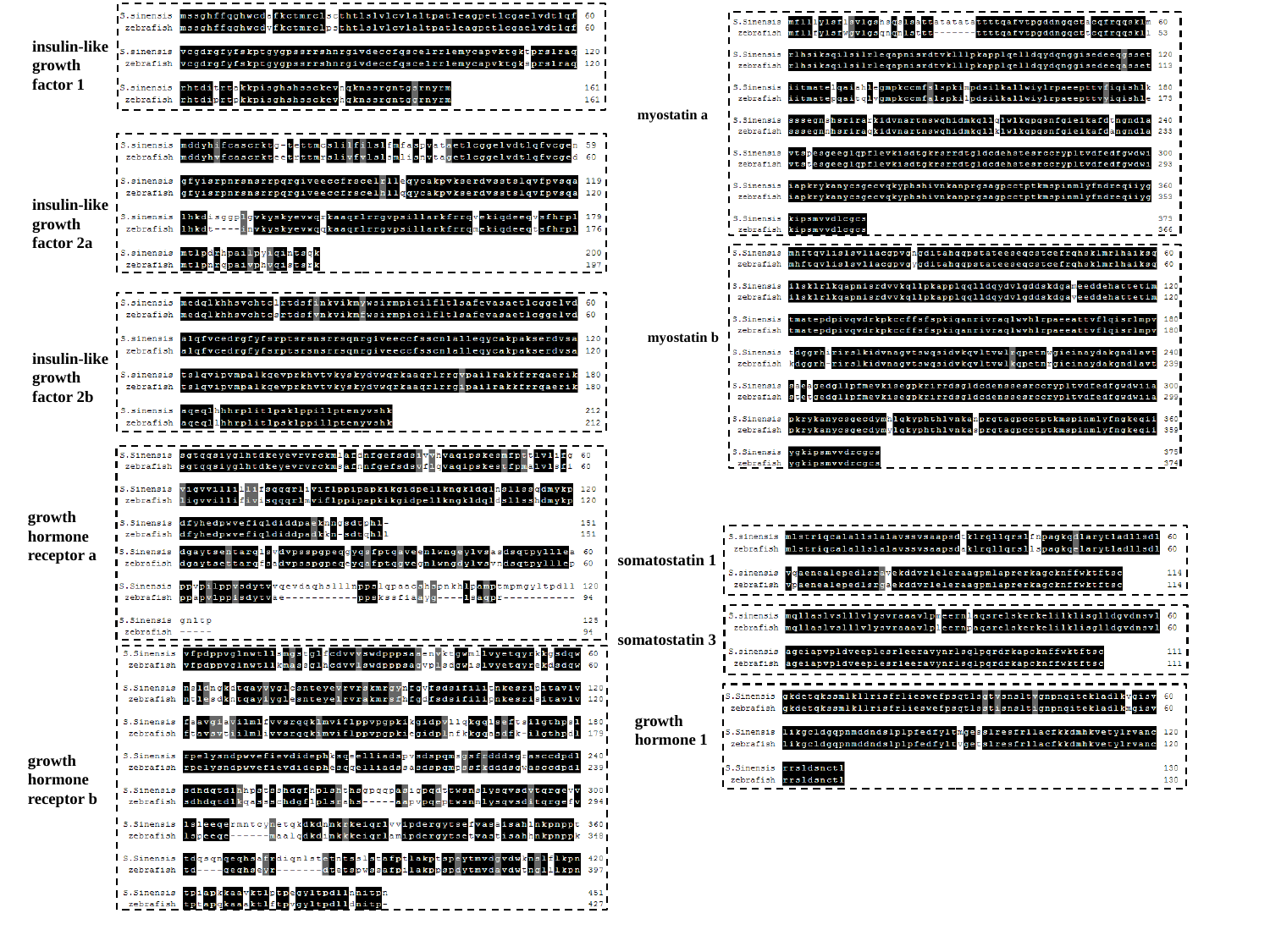

insulin-like growth factor 1
insulin-like growth factor 2a
insulin-like growth factor 2b
myostatin a
myostatin b
growth hormone receptor a
growth hormone receptor b
somatostatin 1
somatostatin 3
growth
hormone 1
