# Supplementary figures and images for: De novo assembly, characterization and annotation for the transcriptome of Sarcocheilichthys sinensis
Source: PLoS One. 2017 Feb 14;12(2):e0171966. doi: 10.1371/journal.pone.0171966 (PMC5308828; doi:10.1371/journal.pone.0171966)

## Slide 1
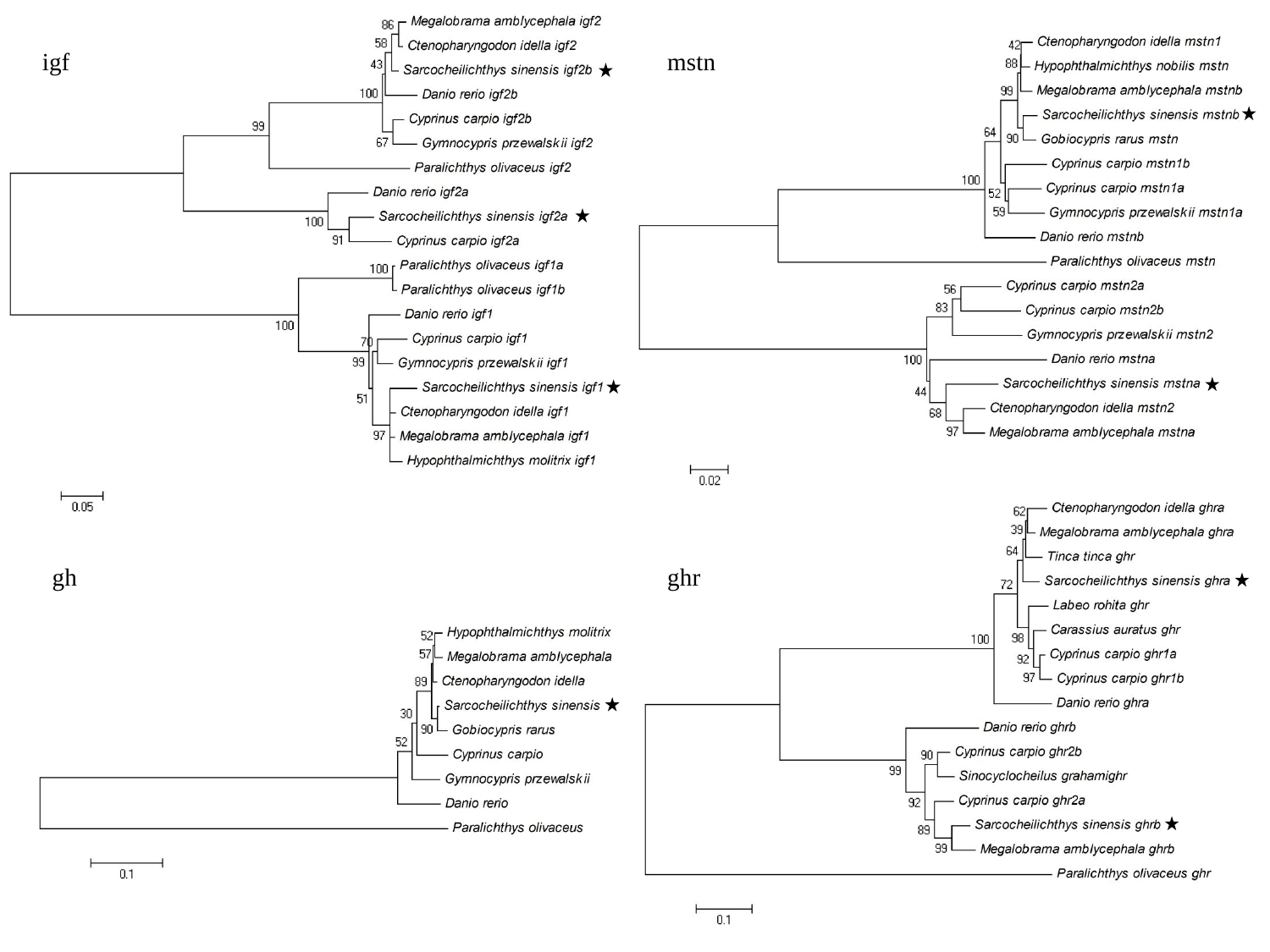

igf
mstn
gh
ghr

Supplement: S2 Fig — GenBank accession numbers for applied sequences were: JN711124.1, NM_001020492.2, AY707317.1, AY170124.1, M27000.1, JF340470.1, M23439.1, JN711123.1 for gh; NM_001083578.1, NM_001111081.1, AY283778.2, GU300104.1, GU300105.1, GU300107.1, GU300108.1, JN896373.1, JN896374.1, XM_016277637.1, AF293417.1, KX082700.1, AB110985.1, AY691177.1 for ghr; NM_131825.2, NM_131433.1, NM_001001815.1, AF332865.1, EU051323.1, JQ398497.1, EF062860.1, HM641129.1, D83272.1, HM755899.1, KC470046.1, AY919608.1, AY919609.1, AJ010602.1, AJ010603.1, AF091454.1 for igf; NM_001004122.2, NM_131019.5, KM874826.1, KM874827.1, JQ065336.1, JQ065337.1, HQ634244.2, KP277104.1, KP277103.1, FJ482232.1, GU014395.1, GU014396.1, GU014397.1, GU014398.1, DQ412048.1 for mstn. (PPTX) [file pone.0171966.s002.pptx]
