# Supplementary material for: De novo assembly, characterization and annotation for the transcriptome of Sarcocheilichthys sinensis
Source: PLoS One. 2017 Feb 14;12(2):e0171966. doi: 10.1371/journal.pone.0171966 (PMC5308828; doi:10.1371/journal.pone.0171966)
Supplement: S1 File — (PDF) [file pone.0171966.s003.pdf]

S1 File. Unigene sequences in transcriptome of *Sarcocheilichthys sinensis* that homologous with 10 zebrafish growth-related genes.

1. Growth hormone

>TR7753_c0_g1
TGGAAAAGATGAAACGCAGAAGAGCTCAATGTTGAAGCTCCTTCGCATCTCTTTCCGC
CTCATTGAGTCCTGGGAGTTCCCCAGCCAGACCCTGAGCGGAACCGTCTCAAACAGCC
TGACCGTCGGGAACCCCAACCAGATCACTGAGAAGCTGGCTGATTTGAAAGTGGGCA
TCAGTGTGCTCATCAAGGGATGTCTGGATGGTCAACCAAACATGGATGATAACGACTC
CCTGCCGCTGCCTTTTGAGGATTTCTACTTGACCATGGGGGAGAGCAGCCTCAGAGAG
AGCTTTCGTCTTCTGGCTTGCTTCAAGAAGGACATGCACAAGGTGGAAACTTACCTGA
GGGTTGCAAATTGCAGGAGATCCCTGGATTCCAACTGCACCCTG**TAG**ATGGCGCCAAT
GTAGTCCTAGTTAAACCTTGTAACACATTTGTGCTTTGATGCAAATCTAAAACCAGTTT
AAATCCTCAAAACCTCCTAAAGCCTAATTATCATCTGGTCTTATATATGCAGGAAATGTC
AACTAGGCATGGCTAGGTCTGTTCTCTAGTTCCCTCCCATATCTAAACCCTACCTACCAC
TATTGTTTTTATTCTTCTCATTGTGGAGTGCTCATAAACTAAAGGCATTAAGGTTTAATCT
GATTTAACATTTCACAGTGGTGCTAAGAAATTTATGGCAACGTTTCAAAAGTGCCCAAA
TCACTTTGACTCTAG

2. growth hormone receptor a

>TR48043_c0_g1
AGTGGCACACAGCAGTCCATCTATGGTCTGCACACTGACAAAGAGTATGAAGTCCGGG
TGCGATGCAAGATGTTAGCCTTTGACAACTTTGGTGAATTCAGTGACAGCATCGTTGTG
CATGTGGCACAGATACCAAGCAAGGAATCAATGTTCCCGACGACGTTGGTGTTGATTTT
TGGAGTGATTGGAGTGGTGATCCTTCTTATCCTCCTCATCTTCTCCCAGCAGCAGAGGT
TGATTGTAATCTTTTTACCGCCTATTCCTGCACCTAAAATTAAAGGCATCGACCCAGAGC
TGCTAAAGAATGGAAAACTTGACCAGCTCAATTCTTTGCTAAGCAGTCAAGATATGTAC
AAGCCGGACTTCTATCATGAGGACCCATGGGTGGAGTTCATCCAGCTGGACATTGATG
ACCCTGCAGAGAAGAACAACGGATCTGATACACCACACCTG

3. growth hormone receptor b

>TR61840_c0_g1
TGTGTTTCCAGACCCACCTGTAGGACTGAATTGGACTCTTTTGAGTATGGGCTCAACTG
GCTTATTCTGTGATGTGGTTGTGAGCTGGGATCCACCTCCGTCTGCTGCAGAGAATGTG
AAGACAGGATGGATGTTGCTGGTGTACGAGACACAGTACAGAAAAAAGGGTTCAGAC
CAGTGGAACTCACTGGATAATGGCAAAGACACGCAGGCATATGTCTATGGTCTCTGCA
GCAACACTGAATATGAAGTCAGAGTCAGGTCAAAAATGAGAGGCTACAATTTTGGTGT
CTTCAGTGACTCCATCTTCATACTCATTACTAACAAAGAATCAAGAATTCCTATTACAGC
TGTGCTTGTCTTCGCTGCAGTTGGTATTGCAGTTATCTTGATGCTCTTTGTAGTATCACG
TCAACAAAAGTTAATGGTGATTTTCCTGCCGCCAGTCCCCGGACCAAAAATCAAAGGG
ATTGACCCTGTGCTTTTACAGAAAGGCCAGCTGAGTGAATTCACATCAATCTTAGGCAC
CCACCCAAGCTTGCGGCCAGAACTGTACAGCAATGATCCATGGGTAGAGTTCATAGAG
GTGGACATCGACGAACCACACAAGAGCCAAGAGGAGCTCCTTATCGCCGACTCTCCG
GTCTCTGACTCACCTCAAATGTCCGGTAGTTTCAGGGATGACGACTCAGGTCGGGCTA
GCTGCTGTGATCCGGACCTGTCAGATCACGATCAAACGGATCTACATCACCCTTCAACC
AGTAGCCACGATGGTTTCCACCCATTGTCGCATACACACTCTGGGCCACAGCAACCTG

CATCCATCGGTCCACAAGACACAACCTGGTCTAACAGCCTTTACTCACAGGTGAGTGA
CGTCACCCAGCGTGGTGAAGTTGTGCTTTCTCTGGAAGAGCAAGAACGGATGAACAC
CTGTTATAATGAGACTCAGAAGGATAAAGACAACAATAAGAGAAAAGAAATCCAACG
GTTGGTGGTGATCCCTGACGAAAGAGGCTACACCTCCGAGTTTGTTGCCAGTGCAATC
AGCGCCCACCTCAATAAACCAAACCCACCTACAACAGACCAATCACAGAATCAAGAA
CAACACAGTGCATTCAGAGACATTCAAAACCTGAGCACTGAGACGAACACGTCCTCT
CTGTCCACTGCATTTCCCACCCTTGCAAAGCCCACCAGTCCAGAGTACACTATGGTAGA
TGGGGTGGACTGGAAAAACAGTCTCTTTCTGAAACCAAATACACCAATCGCCCCAAAG
AAGGCAGCGGTGAAGACTTTGCCCACTCCTGAAGGATACTTGACTCCTGACCTACTCA
ACAACATTACCCC**TAA**CTAAAAGTGGAGTATGTTTGCTCTTTGTGAGATATCTGACATG
GTTAGCCAACACCGTACAAGACGTGTGATCCATGAGGAGGAATCTTGAAATAAAGCCA
ATTCTCAAGCCTTCTCCAGGGGAATGGATGTCATGAAACAAATCTTTTGGCAAGCTAAT
AGTTGGCTGTATAAGGCCTGGAGTGTTTTTTTTTATTCTCCTAATTTAGGGTTAACAGCT
TCAGATGATATTTTGAGCAGACATGGAGCAGAAGTATACATTAAGCTCTCACATGACGT
TGTATAACAGCTATATTGCTGTATTACTGCTCTATATTTTAAGCGCTTTTGTTGTTTGTTTT
CAGAGAACTAACCACCACTATGTGTCTGTGTAAACTGCAAAGTGCCTACTGTAGATTAT
ATTTTAATATCAGTGTTAAAAAAAAGAATGCTACACCTGTAAATCAGAAGGGACTGCTA
TGTTTATGTGAACTGTTACAAGCTTTTGTTCTCAGTGCCATTTTTTTTCCATTAAAAAAC
TGGACTGGCTTTTGTGCCACAATGATGTAAATATGATATATATATAAACATTTTGTTTTCA
CGACAAGCCAACTAAGCTAAAAACAAACAAAAACAGCTTAAACCAGCCTGAAGTGGT
TTCCTGGTCTATCAGTTTGGTATTAGAGTGTTTTGGGCACTTTTTAAGAGAGACCAGCT
AAAACTAATTGGTTTAAGATGTGTGTTTTTTTTTTTTTTTTGTCTA

4. somatostatin 1

>TR56087_c0_g1
GCAAAGCGAAGAGGCAACTTACAGCTTGCGCAGCGCTACCCGACGCATCTCTCTCCTT
TTACTTTTACTCACCAAATCAATACTTTCAAAG**ATG**CTCTCCACGCGTATCCAGTGCGC
ACTGGCGCTCCTGTCTCTCGCGCTCGCCGTCAGCAGCGTCTCAGCAGCACCGTCAGAC
ACCAAACTCCGGCAACTTCTGCAGAGATCTCTCTTCAACCCTGCTGGAAAACAGGATC
TCGCCAGATACACACTTGCAGACTTGCTCTCAGACCTCGTGCAAGCAGAAAACGAGG
CGCTGGAGCCCGAGGATCTGTCTCGCGCCGTGGAGAAAGATGACGTGCGTTTGGAGC
TCGAGCGCGCCGCCGGTCCCATGCTGGCACCTCGCGAGCGCAAAGCCGGATGCAAGA
ACTTCTTCTGGAAAACTTTCACGTCGTGT**TAA**TTCCTCACACCAAAGCGTCTCCTTTG
ACATATTCGTTCTTTTTCTTTTTTTACTCCATATTTCTTTTTCCCGTATTTCACATATTCCTC
ATCCTATCCAAACTGTATATAAGAAAATAAAAGCGGTTATATTTGTCGTTAAACAACGAT
GATAGCTCTCAATGACTATGTTTTTCGAGGGGTATTGATGTGTCATCGGAAAGAATGTTT
TATGGATGTGTGCAAATCTGCTTTTAATTGTACTTTAGCAGATATCACTATTTTTAATTGT
TTGTTTGAATAAAATCTATGTTTCAGAAAAAAAAAAAAAAAAAAAAAAAAAAAAAAA

5. somatostatin 3

>TR19888_c1_g1 （**reverse complementary**）
CAGTAAGGGACTGGAACGAGAGGTATATAAGGACCCTGCCACCTGAAGCGCTCAAAG
ACCAAGATCTCTCCAGCTTTACTCTGCACTAGGAGCTACACAGACTTCGAGGTTCCTGC
TTCGCTTCTCTGACTGACCTTTCTGCGCACTTTAACTTCAACTATTTTGTCAGT**ATG**CA
GCTTTTGGCCAGCCTAGTGTCTCTCTTGCTGGTGCTGTATAGTGTCAGAGCAGCAGCTG
TGCTTCCAATGGAGGAGAGGAACCTGGCACAGAGTAGGGAGCTGAGTAAAGAGCGTA

AGGAGCTGATCCTGAAGCTGATCTCTGGGCTGTTGGATGGAGTAGACAACAGCGTGCT
GGCTGGGGAGATAGCACCGGTCCCCTTGGATGTGGAGGAGCCCTTGGAGTCCCGTCTG
GAAGAACGAGCCGTTTACAACCGGTTATCACAACTGCCACAGCGTGACCGCAAAGCC
CCCTGCAAAAACTTCTTCTGGAAGACCTTCACATCGTGT**TAA**CGGCGCCAGTCCTGCA
ACGATCCCGCGATCCTCCCTGAAACCTGACATGAACTGTGTAGACCTCCACTGTACATA
CCATCTCCAGTGTCTGAAGAAAGGTCTCTGTGGACTTACTCTGACACAGTCTGTTTGTT
AAAGACATTGATGATGATATTTATTTATTTATTTATTTATTTATTGATCGATTAAGCTATTTA
TGGACCTTTGTTGTTTTTCAGCATTAAAAAAAAAGAAGAATAAAGCATGGTTAAGATAGT
TTCTTTTTTGATAAAAGATGTCTCATTTACATTGAGAAAAGCCTCAAATTACAGTATGTT
TACTGACTGATGAATAAATATAATTGATCATGGAGCCAGATGCATTACAGATTGTGTAAT
TGTGCTACGTATAAATGTTTTCCCAAAAGAAGAAATCTCATAAAGGCTATTTGTTACAG
GCTCATCTGGCACTTTCACAAGCCGTGATTGTTTAACACTTAATACATCAGATTAATTGA
TTGACAGTTTGTGTGGTGGTAATTTTCCTTGTCAGTGAATTATATGTAATTGCAGTTGAT
GAGAGGGTGTGCTTGAAGTCACTAAACCCTTGTCTAATAATTATTTGCTGGTAAAATCA
AAGCATTACTAAAATATCTTTCGGTTTGCCTTTTTTTGTTCAACAAATGCAGATGTGATA
CACGTGAGGGAGAAAACATTGTCCGTAAAATACAGGCATTTTCATTTTGAGTCTGTATC
TAAACACTTGTGTTCTTCAATTGCATACATCAAGTTTTTGTCCCATATTAATGGCACTTAT
GCTGCAATCTAGCTCTAACAGAAAAAAAAATCTAAACACTAGGTAGATTGACTTTCCAG
CTGTAAACGTATGCATTAGGCCGTCAGTACAGTTTAATTGTTTGTCCTCCAAGTGGAGG
CATACATGGTTATTCAAACTGTGTCAGCATAAGTACCGAGTTACTTGCGGCAGTGCGTC
ATTGCTCTTGAATGTGAAACTAGTTGGCGTAATGAATGACATGAAAATGCATTGGAAAA
TGCGTGGAGCCATGTGCCGCCTGGATTTCTTATCACTTCTCTACCGTCCTTTCAATGAAT
ATGTCCAAAAAAAAAGTAATGTGGTCATTTGTTGTATTCTGTAAATAATTAGTGTTTAAG
ATAAATGCTAGATATATGGTCACAACACATGCAAACATATGTTATGCTGCTACAGTAAGA
AAAGAAAAAAGTTAATTAATTGTTCAGACTGCTATAGTTATAACTATGTCTCTCTTTAGC
CTCAAAGTTCAAAATGTGAATGACTACATGACTGTTCATGTAAAGATAATAAAAAACAC
CTTGTATGTTGGCAAAATCTGCTGTTTTGTCACCAAAGGCCAA

6. insulin-like growth factor 1

>TR45108_c0_g1（**reverse complementary**）

CTGTAATGTAGATAAATGTGAGGGATTTTCTCTCCAAATCCGTCTCCCGTTCGCTAAATC
TCACTTCTCCACAACGAGCCTGCGCAATGGAACAAAGTCGGAATATTGAGATGTGACA
TTGCCCGCATCTCATCCTCTTTCTCGCTTTTTAATGACTTCAAACAAGTTCATTTTTGCT
GGGCTTTTGCTGGAGACCCATGGGG**ATG**TCTAGCGGTCATTTCTTCCAGGGGCATTGG
TGTGATGCCTTTAAGTGTACCATGCGCTGTCTCTCGTGCACCCACACCCTCTCACTGGT
GCTGTGCGTCCTCGCGTTGACTCCCGCGACACTGGAGGCTGGGCCGGAGACGCTGTG
CGGGGCGGAGCTTGTAGACACGCTGCAGTTTGTGTGTGGAGACCGGGGCTTTTATTTC
AGCAAACCAACAGGATATGGTCCTAGTTCAAGACGATCGCACAACCGCGGCATTGTGG
ACGAATGCTGCTTTCAGAGCTGCGAACTGCGGCGCCTCGAGATGTACTGTGCACCCGT
GAAAACTGGCAAAACTCCACGATCTCTACGAGCGCAACGGCACACAGACATCACCAG
GACAGCAAAGAAACCTATATCTGGACACAGCCACTCTTCCTGTAAGGAGGTTAATCAG
AAGAACTCAAGCCGAGGAAATACAGGGAGCAGAAACTATCGCATG**TAG**AGGACAGC
GAGGGGAACGACTGAGAGAGACAAGCGAAAATGTTGGACAGTGGAAAAAAGGGGAT
GAAAGAAAGACTGGTCTTCCAGGGATGTGCTCCACTGTAAAAGAAAAAAAAAAAAAA
AAAGAAAAAAAAAAAAAAAAGTCTTTCCCC

>TR27626_c1_g1
AAAAAAAAAAAAAAAAAAAAAGAGAGAAAAAAAAAAAAGATACTGGGTGTTTAAGCC
CCATGTTAAGACAAACAAACTTAGTGAAGAAAATCTGATGATTTCCATGCACTGCACCA
TTGCATACTGGGGGAAAATACTAAATTAGCTGCTATAAAGAGTCAAATGATAACTGTTTA
TCCTCCTCGTGTGAACGGTTGCATGTAATGGATGCCGAAAGCTTTTTATAGTGGCAAAA
AAAATGTCAGAGCCCAAATATTGGCCCTACTCTAGTTCGCAGCACCAATTTATGGTCCA
TATTCCACTGAATTCTGATGGACGGCCACTGGAACAGAGGTAATAGGTGGCTATTGGTG
CCCCCCAAACATTGAATTACTTATAATCCTTATCACAATCTGGATCTAGTGGAAGAAAGT
TGCAATATTTTCTCTCAAAAACTAATTTTTGCAGGCAAATGTGCTGCCTTTATGCACAAC
ATTACTGCCAAGAGAGGAGTTTGCAGTGAGATCACAGACAAGAAGTGTTGACAAAGG
CTGTCTAGACAGTTTATACCAGATGGAACCCTTGCAAAACTGAACTACAGCTGCACAG
AAAGATGATGTTTAAAATACAAATCAATCTTTTCTTATTTGATTCATTGTGCAAGACCAG
ATCAAGGGAGATTATACAAAAGACTTATGCAGATGTGCTGAGGTGCTCTAAAGAACATA
TTTAGGATTGATAGAACATGACCTACATCCTGCAAAACACCTTGTGTGTGTTTTTTATTT
TTTATTTTTTAAATTGTGTGTGAGGGATCTTTTCTTCTTTAAAAAAAAAAAAAAAAAAA
AA

7. insulin-like growth factor 2a

>TR39162_c0_g2
CTGAACTTTGAGACAGCCATAAGCATCACTCAAAGAGATGCAACAGACTGGAAATTAA
ACAAAGCAACTCTTTAGAACACAAACGTCGCTTTCCACAAACCGCCTACC**ATG**GATGA
TTACCATATATTCTGTGCATCGTGTCGAAAAACGGGGACAGAAACAACGATGTGCTCAC
TGATACTCTTCATTCTGTCATTGTTCATGTTCGCATCCCCCGTAGCAACGGCGGAGACTC
TTTGCGGTGGAGAACTAGTGGATACTCTGCAGTTCGTGTGTGGAGAAAATGGGTTTTAT
ATCAGCAGGCCGAACAGATCAAACAGCCGCCGTCCTCAGAGAGGAATAGTGGAAGAA
TGCTGCTTTCGGAGCTGCGAGCTGCGTCTGTTGGAGCAGTACTGTGCGAAACCCGTGA
AGTCCGAGCGAGATGTTTCCTCCACCTCGCTGCAGGTCTTCCCAGTGTCGCAGGCTCT
TCACAAGGACATCTCAGGTGGCCCTCTTGGTGTGAAGTATTCCAAATATGAAGTGTGG
CAAAGAAAGGCTGCCCAGAGGTTAAGGAGAGGGGTTCCATCAATCCTGCTTGCACGA
AAGTTCAGGAGGCAGGTGGAGAAAATCCAAGATGAGGAGCAAGTCAGTTTCCACCGC
CCCCTTATGACCCTCCCCGACAGACACCCTGCCATCCTTCCATACATCCAGATCAACAC
GTCCCAAAAA**TGA**TGGCCAGCCTTCCTAAATGGCAATAAACAAGGGATTATAGCCAAG
TCTTGGTCAGTAGAGGCACTTTTTTCATGAATCATACCACTCGTCCACCATCTTTGTCAA
ATCCAGATTATGAACACTCTTGTGTGTTATCAGCCTTCGAAATTCAAGAAGTAAACGTT
GAAAGTGAATGTAATGCGACAGGAGAGATGAATGCAGTCTTGGTGAATATCATGGGGA
TGCCCAGAACTGATGCTGAGGAAGTTGCCAGAACGCCAATGTCAAAAGTCTGCCATCT
GCAAAAGCATTTATGGTTCTGTCTTGCTTGCACCCTATTTTGAATGTCTTATTTTAAGGA
ATGAAATTATCGAGATTAGAGTCCTTTACCTGCACCTTTAAAGTGCCACACCTGTGGCA
TATAGACAGAAGAAAAGTAAGGAAAATGTTGGCTGGCTGTGCAAAGCCAAGGACTGA
TGTTAACAGCACTATTTTTTATGGACTGATAAATATGATAACATTATGGTGCTAACTTTGC
AAAAGGACTCAAAAGGAGTTTGCTAAGGAGTTTTTCCGTTTTAGGAAAAAAAGGAAA
AAAAAGAAGATTGTATTCAAAAGACAATTCAGTAACTCTGGTTGTTTGGAGATCATCCC
AACCGGGAAAAACTGTTTCCATGCTTGGCTGTTCAAGATCTATATTCTGTGCCCTGGAG
AATGAAGGTGAAGCCTTTGCTATTAAACAATATAGCGTTGCAATGGAAGGGAGGAAAC

ATCACAAAATATTGAAGCTGAGGACATCTGCAATCAAGTCGTCTTGGACTTTGGGCAA
GAGCAGTTTCGAGGTGTCACATATTGTACCATAACATTCCTGTTTCCAGTCGGAGTTTC
TGCCATTTTATCTTTATGAATTGTCTTCACTGTTATTGAGACAGTATTTTACACATATAAA
CAAAAGAATGGAACCATCTTTTTAAAAGGAAATTCAGCAATAACCTCTTGCATATTTTC
AACATACACTTGTAGTATTCCAGTGCTTTTTGCTTGAGGGGAAAAAAAAAAAAAAGAA
TACTTGAATATGTATGAATAGTCTCATAAGATGTTGTCATTGACCCTAGTATGACCAGTC
TTGCAAATTGAGCCACCAAACATTTTCTTTAAGTAATGGCACTGTATCAAGGCATTATTT
ATTTTGATATGAAACCCATAAAAATCAGTCCCAATGAGAGATTATGTGCACAACTGACA
GTTTTTGTATATTATCTTTTTTTTAGTTGTTCTGCTTATGTATGTAGATGCTATGTACCAAA
AAATGTCTCTTTTTTTAAGTAAAGTCAATGGAAG
>TR39162_c0_g1
TTTTTTCATGAATCATACCACTCGTCCACCATCTTTGTCAAATCCAGATTATGAACACTC
TTGTGTGTTATCAGCCTTCGAAATTCAAGAAGTAAACGTTGAAAGTGAATGTAATGCGA
CGGAGAGATGAATGCAGTCTTGGTGAATATCATGGGGATGCCCAGAACTGATGCTGAG
GAAGTTGCCAGAACGCCAATGTCAAAAGTCTGCCATCTGCAAAAGCATTTATGGTTCT
GTCTTGCTTGCACCCTATTTTGAATGTCTTATTTTAAGGAATGAAATTATCGAGATTAGA
GTCCTTTACCTGCACCTTTAAAGTGCCACACCTGTGGCATATAGACAGAAGAAAAGTA
AGGAAAATGTTGGCTGGCTGTGCAAAGCCAAGGACTGATGTTAACAGCACTATTTTTA
TGGACTGATAAATATGATAACATTATGGTGCTAACTTTGCAAAAGGACTCAAAAGGAGT
TTGCTAAGGAGTTTTTCCGTTTTAGGAAAAAAAGGAAAAAAAAGAAGATTGTATTCAA
AAGACAATTCAGTAACTCTGGTTGTTTGGAGATCATCCCAACCGGGAAAAACTGTTTC
CATGCTTGGCTGTTCAAGATCTATATTCTGTGCCCTGGAGAATGAAGGTGAAGCCTTTG
CTATTAAACAATATAGCGTTGCAATGGAAGGGAGGAAACATCACAAAATATTGAAGCTG
AGGACATCTGCAATCAAGTCGTCTTGGACTTTGGGCAAGAGCAGTTTCGAGGTGTCAC
ATATTGTACCATAACATTCCTGTTTCCAGTCGGAGTTTCTGCCATTTTATCTTTATGAATT
GTCTTCACTGTTATTGAGACAGTATTTTACACATATAAACAAAAGAATGGAACCATCTTT
TTAAAAGGAAATTCAGCAATAACCTCTTGCATATTTTCAACATACACTTGTAGTATTCCA
GTGCTTTTTGCTTGAGGGGAAAAAAAAAAAAAAGAATACTTGAATATGTATGAATAGT
CTCATAAGATGTTGTCATTGACCCTAGTATGACCAGTCTTGCAAATTGAGCCACCAAAC
ATTTTCTTTAAGTAATGGCACTGTATCAAGGCATTATTTATTTTGATATGAAACCCATAAA
AATCAGTCCCAATGAGAGATTATGTGCACAACTGACAGTTTTTGTATATTATCTTTTTTTT
AGTTGTTCTGCTTATGTATGTAGATGCTATGTACCAAAAAATGTCTCTTTTTTTAAGTAA
AGTCAATGGAAG
8. insulin-like growth factor 2a
>TR19860_c3_g2（**reverse complementary**）
CGGGTTTGGGTATATAATACTTCTCTCACAGAACTTTTGCCTGTCGCCTGATTTTTGGGA
CAGCTCCACACAACATCCTAACACCAACTGGGGAAACTAAACAGACCTTTTCAACCAA
ATACAACAGCACCGCCTTTTTTTGTTTCACATTGTC**ATG**GAGGACCAACTAAAACATC
ATTCTGTTTGCCATACCTGCTTGAGAACAGACAGTTTCATAAATAAGGTAATAAAGATG
TACTGGTCCATACGGATGCCCATATGCATACTGTTTTTAACCCTGTCTGCCTTCGAAGTC
GCTTCAGCTGAGACGTTATGTGGTGGAGAGCTTGTGGACGCGTTGCAGTTTGTGTGCG
AAGACAGAGGCTTCTATTTCAGTCGACCAACTAGCAGGTCGAACAGTCGGCGTTCTCA
AAATCGTGGGATTGTGGAAGAGTGTTGTTTTAGCAGTTGCAACCTAGCTCTACTAGAA
CAGTACTGCGCTAAACCTGCCAAGTCAGAGAGGGACGTTTCAGCCACATCCCTACAGG

TCATCCCGGTGATGCCCGCATTAAAACAGGAAGTCCCAAGAAAGCACGTGACCGTGA
AATATTCCAAATACGACGTGTGGCAACGGAAGGCCGCCCAGAGGCTACGGAGGGGCG
TCCCTGCCATCCTGCGGGCCAAGAAGTTTAGGCGGCAGGCGGAGAGAATCAAGGCCC
AGGAGCAACTGCACCACCACAGGCCTCTCATCACGCTTCCCAGCAAGCTGCCGCCCAT
CCTTCTTCCCACAGAAAACTACGTCAGCCACAAG**TGA**AACCAGGATCATTTGCACAG
AGTCGATAAAAAAAGACTAGGGGATCAAAGCTTTTTGTCTCTGACGTCATTTCTGTGG
CAGTCCTCAACAACCCTTCTTCCTTCCCCACCAGACGTGCTCACACGCTCTTCAAGTTT
CTATTCTTGCTGTTTCGTTCACCAAAAAAAGGCACATCATAAACGAGAGGAACACAAT
TCAGGTGAAGAAGCAAAAGAAAGAGCGAAGAAGTTGCGGAACGCCAGTTCGGAGGA
CATCAGAACGCGAGGAACAGCTCGAGCTAGCATGAAAGAACCCATTCCACTGCATTCT
CCCGAGACAAACGTATCTCTCTTTTAGTCCTTTTTAAATATTAGTTTGCACCTGTAACTAT
AAAGGGACATCCACACTGTAAGGAATTGTTGTAAAATTAGATTCCTGTTCCAGCACCTT
GTAATCACAAATGAAAAGCAGAGAAGAGTCTGCGAATTGCACATCGCCACGGATTACG
TCAAAGTTCTTGTTAAGTAAATAAAAAAGGCACTATTTTTTTATGGACTATGAACATGTA
GCTCAAAAAATGTCATGGTGCTAGCTTTGGGAATGGACTCAAAGGAGAAGGGGTTGA
AAAGCATGTTTTTTTTTTTTCCTTTGAAACTTTATTAAGCTTTCCGTTTTAAGGAAAGTG
TGACTTTGGAAAGAGAAAACAAGAGTGGCTGCGGGGATATCGCCGCATGCAGACGCG
TTCGTAAGACACGCGACGTATGCCTCGGGTCCCGGCGCCGCCGTTGCGATAAAGGAAG
GAGCAGTGTGGGGACGCTGAAGGGATCATGTTCAAATGCCTGGACGTGAAGAACTCG
CCTCCTTTCGAGCGTGGCTGCACAAAACCCCTGCTTTGCACTGCAGGAAAGAGGGAC
ATTGTGACCTTGAAAAAGTGGGATGTTTGTATTTTCTATGTTCTTTTTCGTGAAAACGCT
TCAAGGAAAAAAAAAGAAAAAAAAAAAGCCCAGTTTGGTTTCTGGTACCGTGACATT
CTTGTTGTTGCAAAATAGGCTTTGTGTTTTTGTAACGTTATTTTACAGTTCTGAAGGAA
AAAAAAAAAAGGCACAAATATCAATTCAAAGGGAAAAAACGCAATAATGTCAACTAA
CATATATTTTTAACGTTTATCCGTAGTATTCACATGCTTTTGCCTGAGAAACAAATACTTG
AATATATATATAAATATATATAGAATAAATATGGAAATATATATTATAATTAGTTTCCAGGGA
CATCATGTAATATTCTTTTAGTCTGAGCTGTATCTTGCGTAATGAGCCGCCAAGCTTCTT
TTGTTTGTTTAAGTACAAATATGGGCACTGTATAAAGGCATTATTTATTTTGTTATAAAAT
TATATATTAAAATCAGTCCAAATACAGTAATATACATAGCACTGATGCTCAACTGATGGA
TTTTATTTTATATGCTCTCTTTTTTTTTTTTTTTCATTTTACATTTAAATGCTTT
>TR19860_c3_g1
AAAGCATTTAAATGTAAAATGAAAAAAAAAAAAAAAGAGAGCATATAAAATAAAATCC
ATCAGTTGAGCATCAGTGCTATGTATATTACTGTATTTGGACTGATTTTAATATATAATTTT
ATAACAAAATAAATAATGCCTTTATACAGTGCCCATATTTGTACTTAAACAAACAAAAG
AAGCTTGGCGGCTCATTACGCAAGATACAGCTCAGACTAAAAGAATATTACATGATGTC
CCTGGAAACTAATTATAATATATATTTCCATATTTATTCTATATATATTTATATATATATTCAA
GTATTTGTTTCTCAGGCAAAAGCATGTGAATACTACGGATAAACGTTAAAAATATATGTT
AGTTGACATTATTGCGTTTTTTCCCTTTGAATTGATATTTGTGCCTTTTTTTTTTTTCCTTC
AGAACTGTAAAATAACGTTACAAAAACACAAAGCCTATTTTGCAACAACAAGAATGTC
ACGGTACCAAAAACCAAACTGGGCTTTTTTTTT
>TR19860_c0_g2
GTTATCACTTCTTACATTCTGTGCACGTGGGGATGTTAACGTGAATTCTGCCAGATACG
AAGTTTACAAGAGCTTGCTTGACTTCATCTGAGTTGAACCCTAGTGTGCGGAGCTGAC
GTGCTGTCAGTTGTTCACTGTTTGGTCACATTTTAGTGCATTTTCCTACGTTGGCACACT

TCTGCAGATCAGTCAGTGCAGAAAAGATGCTAAAAGCATCAAGGAAATTCTTTGTAGG
TCTACTAAATCATTCACGGCAATAAAAACACGTACACTTGCACATGACTGAAACATGTT
TGATAAAATGGTTTTTATTGATATTAAAAATGAAAGTACATGAATTGTGCATTTCGGACG
TTCTAACGTGCAGAGGTTAAATCAATTAAAATACACACAGGCCGCGTGGGCTACTGATA
TTAAACTGTTGCCAATGAAAGACAAAAGATCCCTTCATTTTTAGCGCAAAAGGGCGCG
TTTTCCTTACAGCGCCTTTTTCGCACGGAAAGTTAAGTAACCAGTTTGGAAAGAACAA
CAGTGAATGCAGTCCTTGCAGGAATAACATAATAGAAAAATGGCAATGTCTCTGGAAAT
ATGGGATGTTCGAATAATAAAAATAATGATAGTAATTATAATAATTGCACTGCTTCGTCA
CAACTGATATGAATCAGATAAATATTGGATTATCAAGGCAGAGGGATACACCGCAGGCA
CTTAGACCTAAATTTCAAGGTACTAAGCAATACTATAGTATCATTTTTTGACCTTAAGAT
GGGGCTTATACAAGAAACGATGTTGGCTTGTTGTCCTTGTTTGAGAAACCACAAAGAA
AATGGCCTTGTATTGGGTTTTTCACATAAATCCGCTGATATTTCCTGAGTATTAGGGTAG
ATACGTACGGTAGCCCTTCAACAACTGAGATGAAGATAAATGGAGGACTTGAAAAAAC
ACCCCGACCGAAAGGAAATAAGAGGATTTAGGGTGTATTATTGTAAAATGGTGAGATTG
AGGGGACGTATAAACAGTCCACTCTTTGTGACATCATAATGGATCTTTGTGACATCATA
AAGGATCTTTGTGACATCATAATGGATTTTTTTTACTTGGCAACAAGAAAAAAACATTGA
ACCAGGGAAAGGCTCTTCTCATCCCCAAACACATGGCATACGTTAGAAAGCCAAAAAA
TGATGATACAACAATTGAAAACGGCACTAAAGTGGCTTAAACTGTATTGGGTTTGTGTA
CAATTGTGGCATATAGAGACCGGGTATATCCAGATCCATGTGAGGAACGTTTTTTTTTTG
TAGTTGATTTTGTTTTTTTATAAACAGAAATCCATTTGGCAGATTAAAAAGCCATGCGGG
AGGAAGAAAGAGAGAGAGAGAGAGTATTCTGAGTATGTGCCTATTTTTGTATTACAAC
ATGCCTAAGCAATCCAGGCTACAGCTGCTGTTAAAATATTGATTTTAAAATAGCTCTGTG
GCGAACCAAAAGGAAAAAAAGGAAATATTTTTTGGTACAAAGCATCTACAAAAAGCAT
TTACATGTAAAATGAAAAAAAAAAAA
>TR19860_c0_g1
GTTATCACTTCTTACATTCTGTGCACGTGGGGATGTTAACGTGAATTCTGCCAGATACG
AAGTTTACAAGAGCTTGCTTGACTTCATCTGAGTTGAACCCTAGTGTGCGGAGCTGAC
GTGCTGTCAGTTGTTCACTGTTTGGTCACATTTTAGTGCATTTTCCTACGTTGGCACACT
TCTGCAGATCAGTCAGTGCAGAAAAGATGCTAAAAGCATCAAGGAAATTCTTTGTAGG
TCTACTAAATCATTCACGGCAATAAAAACACGTACACTTGCACATGACTGAAACATGTT
TGATAAAATGGTTTTTATTGATATTAAAAATGAAAGTACATGAATTGTGCATTTCGGACG
TTCTAACGTGCAGAGGTTAAATCAATTAAAATACACACAGGCCGCGTGGGCTACTGATA
TTAAACTGTTGCCAATGAAAGACAAAAGATCCCTTCATTTTTAGCGCAAAAGGGCGCG
TTTTCCTTACAGCGCCTTTTTCGCACGGAAAGTTAAGTAACCAGTTTGGAAAGAACAA
CAGTGAATGCAGTCCTTGCAGGAATAACATAATAGAAAAATGGCAATGTCTCTGGAAAT
ATGGGATGTTCGAATAATAAAAATAATGATAGTAATTATAATAATTGCACTGCTTCGTCA
CAACTGATATGAATCAGATAAATATTGGATTATCAAGGCAGAGGGATACACCGCAGGCA
CTTAGACCTAAATTTCAAGGTACTAAGCAATACTATAGTATCATTTTTTGACCTTAAGAT
GGGGCTTATACAAGAAACGATGTTGGCTTGTTGTCCTTGTTTGAGAAACCACAAAGAA
AATGGCCTTGTATTGGGTTTTTCACATAAATCCGCTGATATTTCCTGAGTATTAGGGTAG
ATACGTACGGTAGCCCTTCAACAACTGAGATGAAGATAAATGGAGGACTTGAAAAAAA
CACCCCGACCGAAAGGAAATAAGAGGATTTAGGGTGTATTATTGTAAAATGGTGAGATT
GAGGGGACGTATAAACAGTCCACTCTTTGTGACATCATAATGGATCTTTGTGACATCAT
AAAGGATCTTTGTGACATCATAATGGATTTTTTTTACTTGGCAACAAGAAAAAAACATTG

AACCAGGGAAAGGCTCTTCTCATCCCCAAACACATGGCATACGTTAGAAAGCCAAAAA
ATGATGATACAACAATTGAAAACGGCACTAAAGTGGCTTAAACTGTATTGGGTTTGTGT
ACAATTGTGGCATATAGAGACCGGGTATATCCAGATCCATGTGAGGAACGTTTTTTTTT
GTAGTTGATTTTGTTTTTTTATAAACAGAAATCCATTTGGCAGATTAAAAAGCCATGCGG
GAGGAAGAAAGAGAGAGAGAGAGAGTATTCTGAGTATGTGCCTATTTTTGTATTACAA
CATGCCTAAGCAATCCAGGCTACAGCTGCTGTTAAAATATTGATTTTAAAATAGCTCTGT
GGCGAACCAAAAGGAAAAAAAGGAAATATTTTTTGGTACAAAGCATCTACAAAAAGC
ATTTACATGTAAAATGAAAAAAAAAAAA

9. myostatin a

>TR22102_c0_g2

GGAGAGAGTGGGTAAAGGTGCTGTCCATTCCCAAATGTGTAAACGTCTACAAAGACAC
ATCAGAAGAC**ATG**TTTCTCCTTCTTTATCTGAGCTTTTTGAGTGTGCTGGGGTCACACA
GTCAGAGCCTGAGTGCAACAACTGCAACAGCAACAGCAACAGCAACAACAACAACG
CAAGCATTTGTGACACCTGGAGACGACAACGGCCAGTGTACGGCCTGTCAGTTTAGAC
AGCAGAGTAAACTCATGCGACTGCACTCCATCAAGTCTCAGATTTTGAGCATCCTGCGT
CTAGAACAGGCTCCGAACATCAGTAGAGACACGGTCAAGCTACTCTTACCCAAAGCAC
CTCCACTGCAGGAGCTCCTGGACCAATATGACCAAAATGGAGGCATTAGTGAGGATGA
GGAACAAGGCAGCAGTGAGACCATCATTACCATGGCCACAGAACTGCAAGCCATCGC
CCATCTTGAGGGAATGCCGAAGTGTTGCATGTTCTCACTGAGCCCAAAGATTATGCCCG
ACAGCATCCTGAAGGCCCTGCTCTGGATCTATCTCCGACCAGCTGAGGAGCCAACCAC
AGTCTTCATCCAGATATCTCACCTGAAGTCTTCTTCTGAAGGGAACAGCCACTCAAGAA
TACGCGCACGAAAAATTGATGTGAATGCCCGGACAAATTCCTGGCAGCACATCGATAT
GAAGCAGCTGTTGCAGTTGTGGCTCAAACAGCCCCAAAGTAACTTTGGGATAGAAATC
AAGGCCTTTGACACAAATGGAAATGATCTGGCTGTGACCTCTCCAGAATCTGGAGAGG
AAGGACTGCAACCCTTCCTGGAGGTCAAAATATCAGACACTGGCAAGCGGTCCAGAC
GGGACACCGGCCTTGATTGCGATGAGCATTCCACTGAGTCTCGTTGCTGCCGGTATCCA
CTCACCGTCGACTTCGAGGACTTTGGGTGGGACTGGATAATTGCCCCCAAGCGCTACA
AGGCCAATTACTGCTCCGGTGAATGCGTGCAGAAGTACCCCCACAGTCACATCGTTAA
CAAGGCCAACCCTCGGGGCAGCGCAGGCCCCTGCTGCACACCAACCAAGATGTCACC
CATAAACATGCTGTATTTCAATGACCGTGAGCAGATAATCTATGGAAAAATTCCTTCGAT
GGTTGTAGACCTTTGCGGCTGCTCT**TGA**GCCCAAGGGCTTTTCTAGTCACTACGACAG
CAAGCCCTAACTACTCTGGACCAGAGGGAAGTGAAAACAACCTTTACCTTGTCCCTTA
CAAAATAGTTCCTGCTAAAACAATTAACCACACCAGGTTCCCAACAAGCCAACTTAGC
CAAATCTTTTAGTATTTTCTCATCAACTGTTGCACACAAGGACAGTGGTACCAAGGTGA
CAATGTTTTTTTTTGTTTATGAAGGTATAAGGCTTCCTCTGTGAAAGCATAGAGATGACC
TGATGGGCTTTACCAGCGAGATATCAGAATGTACAAATTGCAAATTGTAAAAAATGAAT
GTCAAAGGGGGTTGTATAGTTGATTATTTATATAAAGTGGCTTATCCCCCAGTGACCCAA
AAACAGGAACACTGACTCAGATACTCACTCAACATGATGTCCAAACAACAGACTTTAG
GAATTCTGCAGTTGGTTCATAGCATATGGGTAGTTGACGAATGATAAGGATAGGAAACT
TTTTAATGCTGTACCAGGTCACAATTTCAATGCAATAGTTTTAAATATTGCTGGAACATG
TCAACGATCCAAAAGCATGCTTCTTATGTTCTTGTCAAAAAATGTCACTGATTAAACTG
ACAATTCAATAGTTATCCTACATAATGTCTTAGGGCTAGAGATGGTCATAGTGATTTCTT
AAAACATGGACTTAATTGTATAGATTCTATGCTTCAAAATCTTGTTGTAGTTTTGATCTG
TTGTACCAAGTAAAATACCTGTTACTGATGTTTCGCGTGAGTTGTTTTCACGTGTCCTTA

AGGGACCTCTTCAATGTTGTCCTCCCGTAACCCTTAACAGAGTGACACCACTGACACC
AATTCAAGCATGTGGAGCACATTATGGCGCTCAAAAGCAACAACCTCATATTCCATGGA
AGCTAGAGCCTCTCCTGTGTTATTTGTTCTCTGGAGGGCTCCCCAGTGTGATGGAGGC
CCCGTGAGGCAACAATACTAGAAGTCTGTCCCCAGGACAATGAGTGCACTTCCAAA

10. myostatin b

>TR58232_c0_g2

CTCTTTAAGAACTGCGAAAGAAGTCCAGCTCTGTCCATTAGGTTTATTGTTGCCAGCTC
AGCCAATCATTGAATCTTACGACACAATAGAGTGGCCAAAGTTGCAGTATAAAAAGCC
TTGCCGAATTTAAGCATGACATCTACTTGTCCGGTGCGTGGTGAGGTTCATTTCCATTG
CAAATCAGATCAAACATAGTAAACATCCTTTAGCACGCTTTGGAAC**ATG**CATTTTACGC
AGGTTTTAATTTCTCTAAGTGTATTAATTGCATGTGGTCCAGTGGGTAATGGAGATATAA
CGGCGCACCAGCAGCCTTCCACAGCCACGGAGGAAAGCGAGCAGTGTTCCACATGTG
AGTTCAGACAACACAGCAAGCTGATGAGACTGCATGCCATCAAGTCCCAAATTCTTAG
CAAACTTCGACTCAAACAGGCGCCAAACATCAGCCGGGACGTGGTCAAGCAGCTGTT
ACCCAAAGCACCGCCTTTGCAACAACTTCTGGATCAGTACGATGTTCTGGGGGATGAC
AGTAAGGATGGAGCTATGGAAGAGGATGATGAACATGCCACCACAGAGACCATAATGA
CCATGGCCACAGAGCGTAAGGATCATCTATTTCAAAGATTCATTCATGTAAATGTATCCT
CTTAATATGGACAAAGAATCTAGGGAATAGCCTACTTGGAGAGACTCTTTACGCAGGCT
TTACGCCCAAGCCAGTAGCACTCACAAGGCAGATATTGTGCATAAAACGCACAGTTAA
GATGTATAATTCTCGTTTCCTGCCTTTTTAAGGCTTTCTGCCCCTTTTTGTCAGCCTTGCT
AAAGCAATTTTACGCACGCTGAACATGGAAGCGCAACAAAAGCACACCAGCGTCGGT
CAGAAGAATTTTTTTCACCTTTACCACACGTGATATAAACTATAAACTGTAGTTTAAATT
GGTTCCCATCTTAGCCAATCTTTTGAGTCAAAATGTGTACTATGTGCAATGCCTTTTATG
TAATGTCTAAATATAGACAGTATATCTGTAGCCGATCCATGCGTTTTTAAAGGTGTAAATA
TTTCCTTCTGCAATGCCGTATGTATATAACATATATACAAGCTATATAGTCATTCTAAGTTT
AACATATAAATGTCTCCTTTTATGTCTGTAATATATTTTATTTTGTGGGCGTAGTCGACGT
TATTTTATTTAAGTTAAATGACAATTTTTTTGTTTCTCTTTTTTTCCCCCAATTACATCAGT
GCGTCATTTTATGGACCGCTTTAATAATTAAACTGTCACTATAAACCAGTCAATCCCGTA
TAATCACTCTGATTTATATTCTCTTCCTTTTTTCAGCTGACCCCATCGTTCAAGTAGATCG
GAAACCGAAGTGTTGTTTTTTCTCCTTCAGTCCGAAAATCCAAGCGAACCGGATCGTA
AGAGCGCAGCTCTGGGTTCATCTGAGACCGGCGGAAGAAGCGACCACAGTCTTCTTA
CAGATATCACGGCTGATGCCCGTTACGGACGGAGGAAGACACATACGAATACGATCCC
TGAAGATAGATGTGAACGCAGGAGTCACGTCTTGGCAGAGTATAGATGTAAAGCAGGT
GCTCACGGTGTGGTTAAGACAACCGGAGACCAACTGGGGCATCGAGATAAACGCGTAT
GACGCGAAGGGAAACGACTTGGCCGTCACCTCAGCCGAGGCTGGAGAGGATGGACTG
CTCCCCTTTATGGAGGTGAAAATCTCAGAGGGCCCAAAGCGAATCCGGAGGGACTCTG
GACTGGACTGCGACGAGAATTCCTCAGAGTCTCGATGCTGTAGATACCCTCTCACTGT
GGACTTCGAGGACTTCGGCTGGGACTGGATTATTGCTCCAAAACGCTATAAGGCGAATT
ATTGTTCGGGAGAATGCGACTACATGCACCTGCAGAAGTATCCCCACACCCATCTGGTG
AACAAGGCCAATCCGCGAGGCACCGCCGGGCCCTGCTGCACCCCCACCAAGATGTCT
CCCATCAACATGCTTTACTTCAACGGCAAAGAGCAGATCATCTACGGAAAGATCCCCTC
AATGGTAGTAGACCGCTGTGGCTGCTCG**TGA**ACCAGTGCCCAGACAGGACTTGATCCA
TCTCAAAGACCCGGACATCTGATCACACCACCCGCCATCCATTATCAGTGCTTTCCGCA
AGACACTGTGCAATAGAAGGACGCTCACTCACTCTCTGGGCACCGCTTCATTTGACTAT

GTTTTTTTTGTCATTTTCCTCTAAATCAGTATTTCTACCACAGGAGTCCAATGTCACATG
GATATACTAAAGCAATGTCTACTGGCTTGACTTGGGAATGGACACTATTGAAATGGACG
ACATTCTCTGCTTTATTTCATGTTTTCACCTTGTCAGAATACTCTCATTAGGATACGCAG
ACAACATACAAAAATTGTATTACACAACCACTCCAAAATGCATTCATTTAGATCTTTACT
GTAACAGCTGTAACCAGTGAGCTTGTTTAAGAAGAGTATCCAAGAGAAACAGAGAGT
GACTCTCGAACATTGAACAGAGCTTGAATACAGTTATCTGACGCAAACTTCCACATACA
CTGCAATAAACACACAGTTAATCATAGCTTTTGTCCACTACTCATCTGTCCAACAGCCCT
ACACACTTGAAGTATTATTGAGATCGGCATTAGGGGAGAGAAGGACTTGAAGCAGAGG
CACTGTGAATGCGGTCTACACATTGAAATGTGTTCGAGACAGAGACATCGGAACTGTA
AGAATGAATGTTAATATCACGCTAACACTCTGTCTTCAAACCACACAGTTTGCACTATG
GCAGACCAATAGAAAGAATTGGTTGCTAAAAATGTTGTAAAAACTGATTTTGATATGTT
TGCTAATTGTATTGTATACTTGCCATTGTTTCCATTAACAGTTGCCTTTTTTAACCACGGT
TAGTACATGTATAAGAACACAAAATAGCAAAAAAGTGTACGATATAAATCTATGTATCTG
TTCAAACAAATAAAGGTGCTTGCTTTATATGTTTAACTCTATTATTTCAAACTGCTTCAC
ACGAAAGGTTATTGAGTTTTTAATCTATAATATCTGGAAACATAATTCTGTACTTGAATG
ATTTAATCCAAGTAATGAAAATCAGTTATATGGTATGGTACCTGATCAGAGCGTTTAAAG
AGGAAATCGTTCCACGTTGTGTCTTTTTATGCACAATGTGTTTGTCCTAAGCAAAGGTG
GATGAAGGGTGTTGTCCGAACTGTGTTAAGAGGTTCCTTTTAGGTTGGATTTACTATCA
TTTGACCTTTTCATTTGTTT
>TR58232_c0_g1
AGTGCCCAGACAGGACTGGATTATTGCTCCAAAACGCTATAAGGCGAATTATTGTTCGG
GAGAATGCGACTACATGCACCTGCAGAAGTATCCCCACACCCATCTGGTGAACAAGGC
CAATCCGCGAGGCACCGCCGGGCCCTGCTGCACCCCCACCAAGATGTCTCCCATCAAC
ATGCTTTACTTCAACGGCAAAGAGCAGATCATCTACGGAAAGATCCCCTCAATGGTAGT
AGACCGCTGTGGCTGCTCGTGAACCAGTGCCCAGACAGGACTTGATCCATCTCAAAGA
CCCGGACATCTGATCACACCACCCGCCATCCATTATCAGTGCTTTCCGCAAGACACTGT
GCAATAGAAGGACGCTCACTCACTCTCTGGGCACCGCTTCATTTGACTATGTTTTTTTT
GTCATTTTCCTCTAAATCAGTATTTCTACCACAGGAGTCCAATGTCACATGGATATACTA
AAGCAATGTCTACTGGCTTGACTTGGGAATGGACACTATTGAAATGGACGACATTCTCT
GCTTTATTTCATGTTTTCACCTTGTCAGAATACTCTCATTAGGATACGCAGACAACATAC
AAAAATTGTATTACACAACCACTCCAAAATGCATTCATTTAGATCTTTACTGTAACAGCT
GTAACCAGTGAGCTTGTTTAAGAAGAGTATCCAAGAGAAACAGAGAGTGACTCTCGA
ACATTGAACAGAGCTTGAATACAGTTATCTGACGCAAACTTCCACATACACTGCAATAA
ACACACAGTTAATCATAGCTTTTGTCCACTACTCATCTGTCCAACAGCCCTACACACTT
GAAGTATTATTGAGATCGGCATTAGGGGAGAGAAGGACTTGAAGCAGAGGCACTGTGA
ATGCGGTCTACACATTGAAATGTGTTCGAGACAGAGACATCGGAACTGTAAGAATGAA
TGTTAATATCACGCTAACACTCTGTCTTCAAACCACACAGTTTGCACTATGGCAGACCA
ATAGAAAGAATTGGTTGCTAAAAATGTTGTAAAAACTGATTTTGATATGTTTGCTAATTG
TATTGTATACTTGCCATTGTTTCCATTAACAGTTGCCTTTTTTAACCACGGTTAGTACATG
TATAAGAACACAAAATAGCAAAAAAGTGTACGATATAAATCTATGTATCTGTTCAAACA
AATAAAGGTGCTTGCTTTATATGTTTAACTCTATTATTTCAAACTGCTTCACACGAAAGG
TTATTGAGTTTTTAATCTATAATATCTGGAAACATAATTCTGTACTTGAATGATTTAATCC
AAGTAATGAAAATCAGTTATATGGTATGGTACCTGATCAGAGCGTTTAAAGAGGAAATC
GTTCCACGTTGTGTCTTTTTATGCACAATGTGTTTGTCCTAAGCAAAGGTGGATGAAGG

GTGTTGTCCGAACTGTGTTAAGAGGTTCCTTTTAGGTTGGATTTACTATCATTTGACCTT
TTCATTTGTTT
